# Morphology of the First Tarsometatarsal Joint and the Incidence of Arthritis and Post-operative Complications

**DOI:** 10.7759/cureus.72605

**Published:** 2024-10-29

**Authors:** Rohid Zamani, Aman Akhtar, Ibrahim I Haq, Alesha Gibbs, Umar N Said

**Affiliations:** 1 Surgery, Leeds Teaching Hospitals NHS Trust, Leeds, GBR; 2 Vascular Surgery, University Hospitals of Coventry and Warwickshire, Coventry, GBR; 3 Trauma and Orthopaedics, George Eliot Hospital, Nuneaton, GBR; 4 Surgery, Leeds Teaching University Hospitals Trust, Leeds, GBR; 5 General Surgery, George Eliot Hospital, Nuneaton, GBR; 6 Trauma and Orthopaedics, Huddersfield Royal Infirmary, Huddersfield, GBR

**Keywords:** anatomy, lapidus, operative complications, osteoarthritis, radiographs, surgery, tarsometatarsal

## Abstract

Introduction

The first tarsometatarsal joint (TMTJ) is often overlooked regarding foot pathology or a secondary measure in most studies, despite its heavy involvement in surgical procedures and foot stability. The primary aim of this study is to assess the effect of the first TMTJ morphology on the incidence of arthritis and post-operative complications following a Lapidus procedure.

Materials and methods

A total of 39 feet/subjects (19 left and 20 right) were assessed by two independent reviewers. The first TMTJ angle and articulating surface shape were measured, and relevant descriptive data was compiled. Statistical analysis was used to analyse variable outcomes via a logistical regression model and inter-rater reliability tests to determine the validity of the methods used.

Results

A statistically significant relationship between first TMTJ angle and incidence of arthritis was revealed but not with articulating surface shape, or between either measure of first​​​​​​​ TMTJ morphology and post-operative complications. Inter-rater reliability tests showed a very strong correlation between inter-rater measurements.

Discussion

The smaller the angle of the first​​​​​​​ TMTJ, the increased incidence of arthritis; therefore, it may be an early sign for clinicians to look for and implement prophylactic interventions sooner. Furthermore, it also signifies that conducting corrective surgeries at this joint will likely have a positive effect on decreasing arthritis pathology. The strong inter-rater reliability findings offer validity to the methods used in this study however can be improved using expert radiographers and AI software.

Conclusion

The first​​​​​​​ TMTJ angle and shape of the articulating surface are both valuable predictors of the incidence of arthritis; however, this study cannot claim that they are good predictors for post-surgical complications. Further research is needed to address the limitations found in this study however it is a valuable initial step in identifying foot pathology early and initiating early management.

## Introduction

The first tarsometatarsal joint (TMTJ) is an often overlooked joint regarding its involvement in the movement of the foot, and hence, its influence on pathologies such as hallux valgus where the first metatarsophalangeal joint (MTPJ) is usually the primary focus [1-4]. Nonetheless, the TMTJ plays a vital role in the support and stability of the foot arches both during stationary weight-bearing and motion [3,5,6].

Due to its vital role, any damage to the TMTJ may predispose an individual to foot pathologies like arthritis, hallux valgus, and pes planus, however, the exact magnitude of this impact is unclear due to the paucity of research in this area, specifically inn relation to arthritis and post-operative complications [7-9]. It is also believed that the morphology of the first TMTJ may influence its impact on foot pathologies. While relevant studies exist, most of them primarily focus on the first TMTJ in terms of general descriptions within the broader population, its mobility, its role in predisposing to hallux valgus deformity, the effects of foot pathology on the smaller joints of the foot, including the first TMTJ, or the methods used to measure the joint’s morphology. [2,4,5,10-12].

To address hallux valgus and arthritis of the First Ray (including the first TMTJ and MTPJ), a Lapidus procedure is performed, during which the joints are fused and realigned using plate and screw fixation to improve function [13]. Despite this, there are very few studies examining the effectiveness of surgery that focus specifically on the fixation of the first TMTJ. Only a limited number of these studies address postoperative complications, and even fewer explore the role of the first TMTJ in these procedures [14]. 

There is a distinct lack of research relating to the morphology of the first TMTJ, especially into the angulation and shape of the articulating surface, and how this relates to the incidence of arthritis and post-operative complications.

This is a novel research topic that has little prior research. Therefore, its results have the potential to give a greater understanding of the clinical relevance of the first TMTJ. There is the potential to affect the way many clinicians look at predisposing factors to problems such as arthritis and allow for earlier intervention to avoid more drastic measures such as surgery. This would therefore alter the treatment focus, especially in cases where we can identify factors that can increase post-operative complications. 

Aim

The primary purpose of this study is to assess the effect the first TMTJ morphology, namely the angle and articulating surface shape, has on the incidence of arthritis in the foot. Furthermore, the secondary aim is to assess how these variables affect the outcome of post-operative complications following a Lapidus procedure to correct Hallux Valgus and other pathologies with the first ray. 

## Materials and methods

Patient identification and eligibility criteria

This is a retrospective study of radiographs from June 2020 to June 2021 from a local database to assess the morphology of the first TMTJ.

This is a quantitative study project that uses both continuous and categorical data to assess the morphology of the first TMTJ. The subjects in this study were over the age of 18, had weight-bearing radiographs, and undergone a Lapidus procedure. Exclusions were no pre- and post-surgical radiographic images to allow for measurement and assessment, radiographs that were non-weight-bearing as this would alter the angles measured, trauma cases due to the complexity of the injury, previous surgery to TMTJ, as this could affect measurements and commentary on incidence measures included in this study. 

Data collection and radiographic analysis

From the local database, basic anonymised demographic information was gathered related to age, sex, confirmed or radiographic evidence of osteoarthritis (pre and post-operatively), and operative details.

Pre-operative, weight-bearing anterior-to-posterior view radiographs were reviewed independently by two researchers (RZ, AA) for the measurement of the first TMTJ angle and the shape of the articulating surface of the first TMTJ. The measuring methods used followed the well-described method of assessing radiographs by Koury et al., 2019 [10], and also seen in other similar studies [15,16]. Inter-rater reliability tests were done to assess the validity of the two reviewers.

As described by Koury et al. [10] the angle was formed by connecting the line formed between the lateral medial borders of the distal aspect of the medial cuneiform and a line running parallel to the lateral border of the medial cuneiform bone. The local radiograph viewing Xero [17] software (Morton and Partners, Cape Town, South Africa) employed by Leeds Teaching Hospitals Trust allowed such measurements to be made in the app. The measurements were combined to find a mean value for the angle of the individual first TMTJ.

Assessment of the first TMTJ articulating surface was also described by Koury et al. [10] and adapted to define the articulating surface as the shape formed by the distal-most end of the medial cuneiform into the following categories: Flat (where there was little to no elevation from the straight line formed by the medial and lateral edges of the distal medial cuneiform bone), Curved (a convex elevation) or Other (any shape that didn’t fit the other categories).

## Results

Thirty-five patients were identified during the data collection, of which nine patients were excluded from the study: four were repeat patients and five lacked radiological imaging. Therefore, a total of 26 patients (15 female and 11 Male) were included with ages ranging from 27 to 82 and a mean age of 63.

Thirty-nine feet (19 left feet and 20 right feet) from the 26 patients were imaged and assessed in relation to the morphology of the first tarsometatarsal joint (TMTJ) and incidence of arthritis (IA) and post-operative complications after a Lapidus procedure (POCL). 

Twenty-seven out of 39 (69%) feet had arthritis in this study and a total of five out of the 31 feet that had a Lapidus procedure recorded complications following surgery (these included infections, malformations, malalignment, and plate/screw instability). 

A Shapiro-Wilkes test for normality was applied to the TMTJ angle measurements, revealing a significance value of 0.109, confirming the data was normally distributed. As the data followed Gaussian distribution, the means could be compared for statistical significance. All statistical analysis was done using SPSS [18].

A descriptive analysis of the left foot revealed the mean angle of the first TMTJ being 19.5 degrees and a range of 35.2 to -9.3 degrees (Table 1). 14 (74%) left feet examined had pre-operative arthritis and 17 (89%) had undergone a Lapidus procedure at the first TMTJ at a later date. 

**Table 1:** Descriptive statistics of left and right feet examined in this study. This table presents the range and mean of the first tarsometatarsal joint (TMTJ) angle measurements, along with the percentage of subjects who exhibited pre-operative arthritis and the percentage who experienced post-operative complications.

Foot	Mean 1st TMTJ angle (degrees)	Minimum 1st TMTJ angle	Max 1st TMTJ angle	Pre-operative arthritis (%)	Lapidus procedure (%)
Left	19.5	-9.3	35.2	74%	89%
Right	15.3	-11.2	38.1	80%	70%

The right foot had a mean angle of the first TMTJ of 15.3 degrees and a range of 38.1 to -11.2 degrees. 16 (80%) right feet had pre-operative arthritis and 14 (70%) had undergone a Lapidus procedure at the first TMTJ at a later date. 

Thirty-one feet altogether had undergone Lapidus procedures (17 left and 14 right) however only 30 of these feet had corresponding images and were included in the statistical analysis. The average mean TMTJ angle for this group was 17.2 degrees with a maximum of 35.2 and a minimum of -11.2. Of this group, 21/30 (70%) had pre-operative arthritis. Only five of the 30 patients had undergone both a left and right foot Lapidus procedure.

Thirty-one feet altogether had undergone Lapidus procedures (17 left and 14 right) however only 30 of these feet had corresponding images and were included in the statistical analysis. The average mean TMTJ angle for this group was 17.2 degrees with a maximum of 35.2 and a minimum of -11.2. Of this group, 21/30 (70%) had pre-operative arthritis. Only five of the 30 patients had undergone both a left and right foot Lapidus procedure.

Incidence of arthritis analysis

To assess the relationship between the morphology of the first TMT joint (angle and articulating shape) and incidence of arthritis (IA) a logistical regression model test was undertaken where IA was the dependent variable and the angle of the first TMTJ and articulating shape were the independent variables. 

The goodness of fit was tested via a Hosmer and Lameshow test revealing a significance value of 0.982 (whereby a value of less than 0.05 would show a poor fit between the predicted values in the logistical model and the observed values). Furthermore, an omnibus test of model coefficients was also done to test goodness of fit showing a significant (P<0.05) value of 0.032. (Table 2).

**Table 2:** Omnibus tests of model coefficients assessing the significance of the logistic regression model predicting the incidence of arthritis based on first TMTJ angle and articulating surface shape. This table presents the results of the omnibus Chi-square tests for the model coefficients at Step 1, examining the incidence of arthritis based on the first tarsometatarsal joint (TMTJ) angle and the articulating surface shape. The Chi-square value, degrees of freedom (df), and significance level (Sig.) are reported for each test: the Step, Block, and overall Model. A Chi-square value of 6.907 with 2 degrees of freedom and a significance level of 0.032 indicates that the model coefficients are statistically significant, suggesting that these anatomical features significantly contribute to the incidence of arthritis.

		Chi-square Value	df	Sig.
Step 1	Step	6.907	2	.032
	Block	6.907	2	.032
	Model	6.907	2	.032

A classification table generated from the regression model revealed an overall percentage of 73.7 in relation to the first TMTJ and articulation shape in predicting the incidence of arthritis. This was positively different than the classification table generated excluding the predictor variables (Table 3). 

**Table 3:** The classification results for the model predicting the incidence of arthritis based on the first tarsometatarsal joint (TMTJ) angle and the joint articulating surface shape. The table shows the observed and predicted counts for arthritis presence (Yes/No) at Step 1, including the number of correctly and incorrectly classified cases. The overall percentage of correctly classified instances is 73.7%, with separate accuracy rates for cases predicted as negative (45.5%) and positive (85.2%). The cut value used for the prediction was 0.500.

Step 1			Predicted		Percentage Correct
			Arthritis (Y/N)		
			N	Y	
Observed	Arthritis (Y/N)	N	5	6	45.5
		Y	4	23	85.2
	Overall Percentage				73.7

The regression model revealed a statistically significant negative effect of the first TMTJ angle on the incidence of arthritis, 0.913 Exp(B) (confidence interval 0.843 - 0.989), and a statistically insignificant positive effect of the first TMTJ articulation shape on the incidence of arthritis 1.4 Exp(B) (confidence interval 0.295 - 6.636) (Table 4).

**Table 4:** Logistic regression results for predicting incidence of arthritis based on first TMTJ angle and joint shape. This table displays the logistic regression coefficients (B), standard errors (S.E.), Wald statistics, degrees of freedom (df), significance levels (Sig.), and exponentiated coefficients (Exp(B)) for the variables in the model (first tarsometatarsal joint (TMTJ) angle and joint shape (Curved or Flat)), predicting the incidence of arthritis. The results indicate that the TMTJ angle is a significant predictor of arthritis (p = 0.026) and Joint shape (Curved vs Flat) was not found to be a statistically significant indicator of arthritis (p = 0.672). Key components of Coefficient (B) = -0.091 for TMTJ, indicating a decrease in the log odds of arthritis as the angle increases. The standard error (S.E) of 0.041 indicates precision and low variability of the coefficient B. The Wald statistic tests whether the coefficient (B) is significantly different from zero, with the TMTJ angle showing a significant effect (Wald = 4.987). The exponentiated B coefficient (Exp(B)) represents the odds ratio, which is the factor by which the odds of arthritis change for a one-unit increase in the predictor. The Exp(B) for the first TMTJ = 0.913 with C.I between 0.843 - 0.989.

Step 1	B	S.E.	Wald	df	Sig.	Exp(B)	95% C.I. for Exp(B) (Lower)	95% C.I. for Exp(B) (Upper)
TMT Joint angle	-0.091	0.041	4.987	1	0.026	0.913	0.843	0.989
Joint shape: Curved or Flat	0.336	0.794	0.179	1	0.672	1.400	0.295	6.636
Constant	2.579	1.103	5.471	1	0.019	13.181		

Outcome of post-operative complications analysis

To assess the relationship between the morphology of the first TMT joint (angle and articulating shape) and incidence of post-operative complications (POC), a logistical regression model test was undertaken where POC was the dependent variable, and the angle of the first TMTJ and articulating shape were the independent variables. As with the logistical regression model for arthritis, articulating shapes were coded as numbers: curved = 1 and flat = 0. 

The goodness of fit was tested via a Hosmer and Lameshow test revealing a value of 0.068 (whereby a value of less than 0.05 would show a poor fit between the predicted values in the logistical model and the observed values). Furthermore, an omnibus test of model coefficients was also done to test goodness of fit showing a non-significant (P>0.05) value of 0.703 (Table 5).

**Table 5:** Omnibus tests of model coefficients assessing the significance of the logistic regression model predicting the incidence of post-operative complications based on first TMTJ angle and articulating surface shape. This table presents the results of the Omnibus Tests of Model Coefficients for the model predicting the incidence of Post-Operative Complications based on the 1st tarsometatarsal joint (TMTJ) angle and joint shape (Curved or Flat). The Chi-square value, degrees of freedom (df), and significance level (Sig.) are reported for each test: the Step, Block, and overall Model. The Chi-square statistic (0.703) tests whether the model, including both the TMTJ angle and joint shape, significantly improves the prediction of arthritis over a baseline model. The degrees of freedom (df) for all tests are 2, and the significance value (Sig.) of 0.703 indicates that the model does not significantly improve the prediction of Post-Operative Complications compared to the null hypothesis. This means that the variables (TMTJ angle and joint shape) do not significantly contribute to predicting Post-Operative Complications in this analysis.

		Chi-square	df	Sig.
Step 1	Step	0.703	2	0.703
	Block	0.703	2	0.703
	Model	0.703	2	0.703

A classification table generated from the regression model revealed an overall percentage of 83.9 in relation to the first TMTJ and articulation shape in predicting post-surgical complications which was no different from the classification table generated which excluded the predictors (Table 6).

**Table 6:** The classification results for the model predicting the incidence of post-operative complications based on the first tarsometatarsal joint (TMTJ) angle and the joint articulating surface shape. This table displays the classification results for the model predicting the incidence of post-operative complications (Y/N) based on the first tarsometatarsal joint (TMTJ) angle and the articulating surface shape. The observed and predicted counts for complications are shown at Step 1. The model correctly classified 100% of cases with no complications (N) but failed to correctly classify any cases with complications (Y). The overall percentage of correctly classified cases is 83.9%. The model uses a cut value of .500, meaning that predicted probabilities above 0.5 were classified as "Y" (complications), while those below 0.5 were classified as "N" (no complications).

Observed			Predicted		Percentage Correct
			Arthritis (Y/N)		
			N	Y	
Step 1	Arthritis (Y/N)	N	26	0	100.0
		Y	5	0	0.0
	Overall Percentage				83.9

Looking at the individual variables for impact on the incidence of post-operative complications in the model revealed a statistically insignificant expected change in outcome (Exp(B)) of 0.689 (CI 0.081 - 5.858) for a unit change in joint articulating shape and a statistically insignificant Exp(B) of 0.966 (CI 0.891 - 1.048) for a unit change in TMTJ angle (Table 7).

**Table 7:** Logistic regression results for predicting post-operative complications based on first TMTJ angle and joint shape. This table presents the logistic regression results for predicting post-operative complications based on joint shape (Curved or Flat) and the first tarsometatarsal joint (TMTJ) angle. The B coefficients, standard errors (S.E.), Wald statistics, degrees of freedom (df), significance levels (Sig.), and odds ratios (Exp(B)) are provided for each variable. Joint shape (Curved or Flat) has a B of -0.373 and an Exp(B) of 0.689, but this is not statistically significant (p = 0.733). The wide confidence interval (0.081 - 5.858) suggests a high degree of uncertainty in the estimate. TMT Joint angle has a B of -0.034 and an Exp(B) of 0.966, indicating a slight decrease in the odds of post-operative complications with increasing angle, though this effect is also not significant (p = 0.407). The Constant has a B of -0.868 with an Exp(B) of 0.420, representing the baseline odds of complications when all predictors are set to zero.

Step 1	B	S.E.	Wald	df	Sig.	Exp(B)	95% C.I. for Exp(B) (Lower)	95% C.I. for Exp(B) (Upper)
Joint shape: Curved or Flat(1)	-0.373	1.092	0.116	1	0.733	0.689	0.081	5.858
TMT Joint angle	-0.034	0.042	0.688	1	0.407	0.966	0.891	1.048
Constant	-0.868	1.158	0.562	1	0.453	0.420		

Inter-rater reliability analysis

An Intra-class correlation coefficient test was done for the first TMTJ angle values measured by RZ and AA for both left and right feet to assess inter-rater reliability. Both Left and right foot measurements showed a statistically significant strong positive correlation between RZ and AA. (Table 8).

**Table 8:** Intra-class correlation of left and right foot first TMTJ angle measurements. This table presents the intra-class correlation coefficients (ICC) for the first tarsometatarsal joint (TMTJ) angle measurements of the left and right feet. The results show high inter-rater reliability for both feet, with an ICC of 0.958 for the left foot (95% CI: 0.893–0.984) and 0.979 for the right foot (95% CI: 0.948–0.992). Both measurements were statistically significant (p < 0.05), indicating consistent and reproducible measurements of the first TMTJ angle across subjects.

	Intra-class correlation	95% Confidence interval	P Value (P<0.05 = significant)
Left foot 1^st^ TMTJ angle	0.958	0.893 – 0.984	<0.05
Right foot 1^st^ TMTJ angle	0.979	0.948-0.992	<0.05

Articulating surface assessments by RZ and AA were coded into a binomial variable (Curved =1 Flat= 0) and a Cohen’s Kappa test was conducted for inter-rater reliability. Both left and right feet surface assessments by RZ and AA showed a statistically significant Kappa score. The assessment of the left foot articulating surface shape showed moderate agreement (Kappa score = 0.568) while the right foot articulating surface shape showed strong agreement (Kappa Score = 1) between RZ and AA (Table 9).

**Table 9:** Cohens Kappa test for measure of agreement between the articulating surface shape of left foot and right foot first TMTJ. This table presents the Cohen's Kappa scores, measuring the agreement between assessors of the articulating surface shape of the 1st tarsometatarsal joint (TMTJ) between the left and right feet. The Kappa score for the left foot is 0.568 (p = 0.013), indicating moderate agreement, while the right foot shows perfect agreement with a Kappa score of 1 (p < 0.05). These values suggest that there is a high level of consistency in categorising the articulating surface shape in both feet, with statistical significance for both measurements (p < 0.05).

	Measure of agreement (Kappa score -1 – +1)	P Value (P<0.05 = significant)
Left Foot Articulating Surface Shape	0.568	0.013
Right Foot Articulating Surface Shape	1	<0.05

## Discussion

Both the angle and articulating surface shape of the first TMTJ are shown to be strong predictors of the incidence of arthritis. The logistical regression model which combined the two predictors resulted in a 73.5% confidence in its outcome, a higher percentage than when these predictors were excluded. This means that the angle and articulating shape of the first TMTJ are both reliable and good predictors of the incidence of arthritis.

The separate variables as seen in Table 4 had revealed that the first TMTJ angle was statistically significant in predicting the weak negative relationship between angulation and incidence of arthritis, with a value of 0.913. This suggests that the smaller the angulation of the first TMTJ the higher the incidence of arthritis. The narrow confidence interval that doesn’t span 1 (0.843-0.989) further supports the significance of this result.

The joint shape was revealed to have a positive relationship in predicting arthritis with an Exp(B) value of 1.4 which, due to the coding of this variable where a curved articulating surface is coded as a higher number than flat (curved =1 and Flat = 0), suggests that the more curved the articulating surface the higher the incidence of arthritis in this joint. However, this variable was found to be statistically insignificant (P>0.05), further demonstrated by the wide confidence interval that spanned from 0.295 to 6.636, undermining its reliability as a predictor for arthritis. 

Despite the lack of significance in the analysis, the results addressing the impact of the first TMTJ morphology are a valuable first step in guiding further research, especially in light of the lack of research into this relationship. A study into the angle at different points in time before there is evidence of arthritis would be most optimal in finding out how early changes can be noted and identifying time points that would be most beneficial to correct the angulation through physio, orthotics, and minor interventions to minimize the risk of arthritis [9,19]. 

When conducting a logistical regression model using the first TMTJ angle and Articulation surface to predict the incidence of post-operative complications the classification table showed an 83.9% confidence score. This displays a strong relationship between the predictors and outcome; however, this value was no different than what was observed in the classification table excluding the predictors; hence, the first TMTJ angle and articulating surface shape are poor predictors for incidence of postoperative complications.

Furthermore, the regression model had a poor goodness of fit value both in the omnibus tests of model coefficients and the Hosmer and Lameshow test which means there was too much variability for the model to reliably assess the strength of the predictor variables for post-operative complications. Nonetheless, this may be due to the small cohort size for patients with post-operative complications. Therefore the data set was insufficient to draw these conclusions and further targeted research is needed to sufficiently comment on this relationship. 

This was highlighted in Table 7, which showed that both TMTJ angle (Exp(B) 0.689, CI: 0.891 - 1.048 ) and articulating surface (Exp(B) 0.966, CI: 0.081 - 5.858) had insignificant (P>0.05) expectant change in outcome Exp(B) values, evidenced further by the wide confidence intervals spanning zero in both individual variables which means that it’s unclear whether the true effect of either variable on the incidence of post-operative complications is negative or positive.

While no relationship was found between the morphology of the first TMTJ and post-operative complications, it should be noted that only five subjects out of 31 presented with complications post-operatively which substantially limits the validity of these findings. Whilst general impressions for the general population could be applied, there is no scope for identifying individual complications with this cohort. For this to be achieved, it would be necessary to conduct the study on a larger cohort comprising patients with post-operative complications to assess whether they share any commonalities in their first TMTJ morphology, as well as a control group that had the Lapidus procedure but had no noted complications to compare with.

There was strong inter-rater reliability in both the assessment of articulating surface shape and angle of the first TMTJ. There is a moderate to strong correlation between RZ and AA and the methods used have been replicated in other studies to mitigate for lack of experience. This shows that there is reliability in the measurements taken and that the use of them in this study is valid. However, it should be noted that both assessors were not expert radiologists or specialists in foot and ankle orthopaedic surgery. The inter-rater reliability can be improved and made more reliable with the use of experts and by increasing the number of assessors to decrease the likelihood of errors or false positive results. Trained professionals such as radiographers may be better suited for collecting these measurements.

Limitations

A notable limitation identified was that many other ways to measure the morphology of the first TMTJ that were not explored in this study. For example, this study only looked at the foot in the antero-posterior view, excluding lateral and oblique radiographs. The first TMTJ is a 3D structure and to fully comment on its morphology, measurements should be taken from different views to give a more comprehensive analysis of the hypothesis [15,20]. To improve this study multiple measuring methods could be applied to offer more validity, especially if the analysis using multiple methods also agrees with the findings in this study.

There are other factors that could also affect the images assessed such as weight which will not have been addressed in this study as the X-rays were taken from clinical settings. For more comprehensive adjustments for this limitation, radiographs should be taken in a controlled lab where environmental factors affecting the resulting angle, such as weight through the foot, can be mitigated [21-23].

## Conclusions

The results of this study show that the first TMTJ angle and shape of the articulating surface are both valuable in predicting the incidence of arthritis. This has the benefit of allowing early preventative interventions, alongside the identification of candidacy for the Lapidus procedure.

Whilst the relationship between predictor variables and post-operative outcomes was insignificant, this lack of relationship may be accounted for by the small sample size available of patients experiencing post-operative morbidity, and thus may still warrant further research with a larger cohort. This study is a valuable first step in identifying how the often-overlooked first TMTJ may be impacting the incidence of arthritis, and additionally impacting the efficacy of corrective treatment strategies, hence, it can form a reliable template for further research into this widely unexplored topic.
